# Patients with atrial fibrillation and outcomes of cerebral infarction in those with treatment of warfarin versus no warfarin with references to CHA_2_DS_2_-VASc score, age and sex - A Swedish nationwide observational study with 48 433 patients

**DOI:** 10.1371/journal.pone.0176846

**Published:** 2017-05-04

**Authors:** Tommy Andersson, Anders Magnuson, Ing-Liss Bryngelsson, Ole Frøbert, Karin M. Henriksson, Nils Edvardsson, Dritan Poçi

**Affiliations:** 1 Department of Cardiology, Faculty of Health, Örebro University, Örebro, Sweden; 2 Clinical Epidemiology and Biostatistics, School of Medical Sciences, Örebro University, Örebro, Sweden; 3 Department of Occupational and Environmental Medicine, Örebro University, Örebro, Sweden; 4 Department of Medical Science, Uppsala University, Uppsala, Sweden; 5 Sahlgrenska Academy at Sahlgrenska University Hospital, Göteborg, Sweden; University of Bologna, ITALY

## Abstract

**Aims:**

There is controversy in the guidelines as to whether patients with atrial fibrillation and a low risk of stroke should be treated with anticoagulation, especially those with a CHA_2_DS_2_-VASc score of 1 point.

**Methods:**

In a retrospective, nationwide cohort study, we used the Swedish National Patient Registry, the National Prescribed Drugs Registry, the Swedish Registry of Education and the Population and Housing Census Registry. 48 433 patients were identified between 1 January 2006 and 31 December 2008 with incident atrial fibrillation who were divided in age categories, sex and a CHA_2_DS_2_-VASc score of 0, 1, 2 and ≥3 and they were included in a time-varying analysis of warfarin treatment versus no treatment. The primary end-point was cerebral infarction and stroke, and patients were followed until 31 December 2009.

**Results:**

Patients with 1 point from the CHA_2_DS_2_-VASc score showed the following adjusted hazard ratios (HR) with a 95% confidence interval: men 65–74 years 0.46 (0.25–0.83), men <65 years 1.11 (0.56–2.23) and women <65 years 2.13 (0.94–4.82), where HR <1 indicates protection with warfarin. In patients <65 years and 2 points, HR in men was 0.35 (0.18–0.69) and in women 1.84 (0.86–3.94) while, in women with at least 3 points, HR was 0.31 (0.16–0.59). In patients 65–74 years and 2 points, HR in men was 0.37 (0.23–0.59) and in women 0.39 (0.21–0.73). Categories including age ≥65 years or ≥3 points showed a statistically significant protection from warfarin.

**Conclusions:**

Our results support that treatment with anticoagulation may be considered in all patients with an incident atrial fibrillation diagnosis and an age of 65 years and older, i.e. also when the CHA_2_DS_2_-VASc score is 1.

## Introduction

Treatment with an anticoagulant in patients with atrial fibrillation (AF) decreases the risk of cerebral infarction; the challenge consists of balancing between protection against thromboembolic diseases and complications of bleeding, and risk scores have been under development for almost 20 years [1–3]. Guidelines recommend the CHA_2_DS_2_-VASc score, which is the most commonly used risk score in clinical practice today [3–4]. According to the CHA_2_DS_2_-VASc score, anticoagulant therapy is recommended or considered for patients with 2 or more points while those with a 0 point should not be treated [3]. However, the 1 point group constitutes a gray zone with a reported annual incidence of stroke and transient ischemic attack (TIA) between 0.2% and 6.6% in different studies [5–11]. Some studies have indicated that, among patients with AF and 1 point in the CHA_2_DS_2_-VASc score, an age between 65 and 74 years is at the highest risk for stroke and TIA [5,8,12]. Our aim was, in a nationwide cohort of patients with incident AF with and without warfarin therapy, to compare the risks of cerebral infarction and stroke according to CHA_2_DS_2_-VASc score, age and sex.

## Materials and methods

We conducted a nationwide, retrospective cohort study using the Swedish National Patient Registry, the National Prescribed Drugs Registry, the Swedish Register of Education and the Population and Housing Census Registry, and the study cohort was identified by the epidemiological centre at the Swedish National Board of Health and Welfare and Statistics Sweden. The Swedish National Patient Registry has a >99% coverage of hospital diagnoses from 1987 and onwards, and the diagnoses have a positive predictive value of 85–95% [13]. The validity of the registry is high and has therefore been recommended for use in epidemiological studies by the National Board of Health and Welfare [13–14]. Information about medication was obtained from the National Prescribed Drugs Registry, which started 1 July 2005. To increase the probability that patients did not take warfarin before the study period began, we applied a washout period between 1 July 2005 and six months afterwards. Thus, patients with incident AF between 1 January 2006 and 31 December 2008 were eligible. AF was defined according to the International Classification of Diseases (ICD): 427 D (DA, DB, DC, DD, DW) in ICD 9 (1987–1996) and I 48, I48.9 and I 48.9 (A, B, C, D, E, F, P, X) in ICD 10 (1997-). According to the CHA_2_DS_2_-VASc score, patients could receive a maximum of 9 points: age 65 to 74 years (1 point) and ≥75 years (2 points), female sex (1 point) and diagnoses from ICD 9 and ICD 10: congestive heart failure 428 and I 50 (1 point), hypertension 401–405 and I 10–14 (1 point), diabetes mellitus 250 and E 10–14 (1 point), cerebral infarction 433–434 and I 63–64 (2 points), TIA 435 and G 45 (2 points) and vascular disease 410–414, 440, 443.9 and I 20–25, I 70, I 73.9 (1 point) from 1987 and until 30 days after the date of diagnosis of incident AF. The educational background was obtained from the Swedish Register of Education and the Population and Housing Census Registry by Statistics Sweden.

Warfarin was the only registered oral anticoagulant in Sweden during our study period, and phenprocoumon could be prescribed [5]. During the years of this study, acetylsalicylic acid (ASA) was recommended for patients with 0 and 1 point based on the CHADS_2_ score [1]. Recent studies do not support ASA in patients with AF, and current guidelines do not recommend it for protection of cerebral infarction; we therefore did not consider ASA to be analyzed [3,15,16].

In Sweden, all inhabitants have a unique personal identification number, all have equal access to health care and hospital services, and hospitals are required to record all discharge diagnoses. This provides the possibility to record and track all non-emigrated patients in clinical registries and examine morbidity and mortality in the entire Swedish population. This study complied with the Declaration of Helsinki, and the study protocol was approved by the Regional Ethical Review Board in Uppsala, Sweden (Dnr 2009/273).

### Study cohort

**Fig 1:**
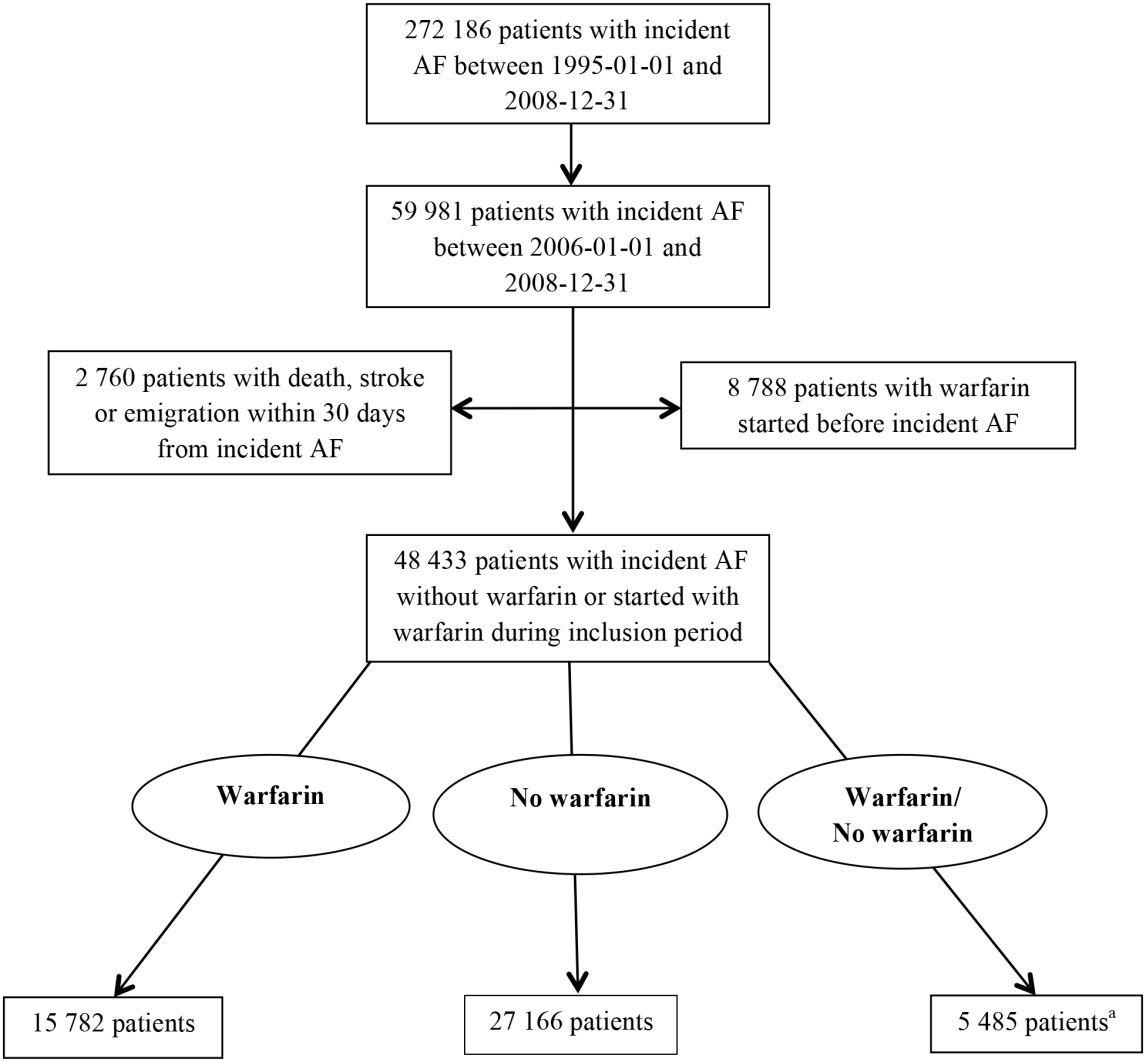
Flowchart. All patients were followed until an event of cerebral infarction, stroke, death, emigration or end of study at 31 December 2009. Patients in warfarin/no warfarin could belong to both the exposed and non-exposed arm. ^a^Patients received warfarin after 30 days or more from incident atrial fibrillation or irregular withdrawal during the study period or until occurrence of an endpoint.

In total, there were 272 186 patients with incident AF between 1987 and 2008 and, of those, 59 981 had a diagnosis of AF during the inclusion period between 1 January 2006 and 31 December 2008 (Fig 1). We excluded 11 548 patients who had warfarin before the diagnosis of AF or had died, emigrated or suffered a stroke within 30 days after diagnosis. The remaining 48 433 patients were categorized into three groups: 1) warfarin treatment started within 30 days and regular withdrawal, 2) no warfarin and 3) warfarin treatment started after 30 days or more or irregular withdrawal. These groups consisted of 15 782, 27 166 and 5 485 patients, respectively (Table 1). Our definition of regular withdrawal was that warfarin had been collected at a pharmacy at least once a year after the date of incident AF. Over- and under-treatment are well known, and some patients were treated regularly with warfarin contrary to contemporary guidelines [17]. Although treatment should be started instantly when the indication is clear, we applied a cut-off at 30 days so that the necessary diagnostics, for example echocardiography or newly discovered diabetes mellitus, were completed to determine the CHA_2_DS_2_-VASc score.

**Table 1:** Baseline characteristics.

	1.Warfarin	2.No warfarin	3.Warfarin/ No warfarin^a^	Total
**Patients**^b^	15 782 (42.4)	27 166 (45.1)	5 485 (45.6)	48 433 (44.3)
**Age (years)**				
Mean±SD	71.5±9.7	71.7±12.5	70.7±10.2	71.5±11.4
Women mean±SD	74.1±8.2	74.4±10.8	73.0±9.1	74.1±9.8
Men mean±SD	69.5±10.3	69.5±13.4	68.8±10.7	69.5±12.1
**Year, diagnosis of AF**				
2006	4 646	8 952	2 315	15 913
2007	5 304	8 990	1 838	16 130
2008	5 834	9 224	1 332	16 390
**Age categories**^b^				
<65 years	3 610 (23.9)	6 551 (24.1)	1 374 (25.1)	11 535 (23.8)
65–74 years	5 055 (32.0)	6 315 (23.2)	1 787 (32.6)	13 157 (27.2)
75–85 years	7 117 (45.1)	14 300 (52.6)	2 324 (42.4)	23 741 (49.0)
**CHA**_**2**_**DS**_**2**_**-VASc score**^c^				
0	1 005 (6.4)	2 547 (9.4)	421 (7.7)	3 973 (8.2)
1	1 885 (11.9)	3 251 (12.0)	798 (14.5)	5 934 (12.3)
2	2 855 (18.1)	4 369 (16.1)	1 065 (19.4)	8 289 (17.1)
≥3	10 037 (63.6)	16 999 (62.6)	3 201 (58.4)	30 237 (62.4)
*3*	*3 369 (21*.*4)*	*5 374 (19*.*8)*	*1 202 (21*.*9)*	*9 945 (20*.*5)*
*4*	*3 069 (19*.*3)*	*4 945 (18*.*2)*	*1 018 (18*.*6)*	*9 032 (18*.*6)*
*5*	*2 050 (13*.*0)*	*3 410 (12*.*6)*	*547 (10*.*0)*	*6 007 (12*.*4)*
*6*	*1 046 (6*.*6)*	*2 056 (7*.*6)*	*293 (5*.*3)*	*3 395 (7*.*0)*
*7*	*398 (2*.*5)*	*878 (3*.*2)*	*108 (2*.*0)*	*1 384 (2*.*9)*
*8*	*90 (0*.*6)*	*276 (1*.*0)*	*30 (0*.*5)*	*396 (0*.*8)*
*9*	*15 (0*.*1)*	*60 (0*.*2)*	*3 (0*.*1)*	*78 (0*.*2)*
**CHA**_**2**_**DS**_**2**_**-VASc diseases**^c^				
Hypertension	7 091 (44.9)	11 588 (42.7)	2 411 (44.0)	21 090 (43.5)
Ischemic heart disease	4 300 (27.2)	8 925 (32.9)	1 687 (30.8)	14 912 (30.8)
Heart failure	4 446 (28.2)	5 559 (20.5)	1 035 (18.9)	11 040 (22.8)
Diabetes mellitus	2 303 (14.6)	4 474 (16.5)	783 (14.3)	7 560 (15.6)
Myocardial infarction	1 914 (12.1)	4 542 (16.7)	858 (15.6)	7 314 (15.1)
Stroke	2 231 (14.1)	3 305 (12.2)	466 (8.5)	6 002 (12.4)
TIA	906 (5.7)	1 019 (3.8)	185 (3.4)	2 110 (4.4)
Atherosclerosis	287 (1.8)	785 (2.9)	110 (2.0)	1 182 (2.4)
Vascular disease	175 (1.1)	394 (1.5)	74 (1.3)	643 (1.3)
**Other diseases**^c^				
Tumores	2 291 (14.5)	7 150 (26.3)	988 (18.0)	10 429 (21.5)
COPD	909 (5.8)	2 236 (8.2)	324 (5.9)	3 469 (7.2)
Chronic renal failure	235 (1.5)	869 (3.2)	115 (2.1)	1 219 (2.5)
**Education**^c^				
1.Primary education <9 years	6 246 (39.6)	11 127 (41.0)	2 027 (37.0)	19 400 (40.1)
2.Primary education ≥9 years	1 201 (7.6)	2 034 (7.5)	419 (7.6)	3 654 (7.5)
3.Upper secondary education <3 years	4 048 (25.6)	6 465 (23.8)	1 386 (25.3)	11 899 (24.6)
4.Upper secondary education ≥3 years	1 510 (9.6)	2 635 (9.7)	550 (10.0)	4 695 (9.7)
5.Post-secondary education <3 years	1 083 (6.9)	1 830 (6.7)	443 (8.1)	3 356 (6.9)
6.Post-secondary education ≥3 years	1 349 (8.5)	2 235 (8.2)	524 (9.6)	4 108 (8.5)
7.Post-graduate	98 (0.6)	201 (0.7)	49 (0.9)	348 (0.7)
8.Unknown	247 (1.6)	639 (2.4)	87 (1.6)	973 (2.0)

SD indicates standard deviation; AF, atrial fibrillation; COPD, chronic obstructive pulmonary disease; and TIA, transitoric ischemic attack

^a^Patients received warfarin after 30 days or more from incident atrial fibrillation or irregular withdrawal during the study period until 2009-12-31 or occurrence of an endpoint

^b^Women in percent

^c^Proportion in percent

### Time-varying exposure analysis

To include all eligible patients and to decrease the risk of treatment misclassification, we used time-varying exposure analysis. This means that patients with irregular warfarin treatment or who had started warfarin after 30 days or more from incident AF are included in both the exposed and non-exposed arms at different times depending on whether warfarin treatment is on-going (Table 2). Patients with warfarin treatment started within 30 days of incident AF were analyzed in the exposed arm and those without warfarin in the non-exposed arm during the study period or until an endpoint.

**Table 2:** Study groups and possible exposures to warfarin in the time-varying analysis.

Study groups	n	%	Warfarin	No warfarin
1.Warfarin regularly and started within 30 days, exposed	15 782	32.6	X	
2.No warfarin, non-exposed	27 166	56.1		X
3.Warfarin irregularly or started after 30 days or more	5 485	11.3	X	X
Total	48 433	100		

In the time-varying analysis, patients with warfarin with irregular withdrawal or who started after 30 days or more are included in the exposed or non-exposed arms at different times depending on whether warfarin treatment was on-going.

### Outcomes

The primary outcome was a diagnosis of cerebral infarction and stroke. The classifications of diagnosis from the ICD 10 were: I 63 (0, 1, 2, 3, 4, 5, 6, 8, 9) and I 64. The secondary outcome was cerebral bleeding I 60-I 62. We used a quarantine period to reduce an overestimate of outcomes, and the follow-up began 30 days after the date of incident AF [7]. The follow-up time ended on the date of outcome, death, emigration or the end of the study on 31 December 2009, whichever occurred first.

### Statistical analysis

Continuous variables are presented as mean ± SD and categorical as percentages. Cox regression was used to evaluate the relative risk of cerebral infarction and stroke to warfarin treatment as a time-varying exposure, adjusted for potential confounders. Adjustment was made for age, year of AF diagnosis, sex, neoplasm (140–239 and C 00-D 48), chronic obstructive pulmonary diseases (COPD) (496 and J 44), chronic renal failure (585 and N 18) and education. Neoplasm, COPD and chronic renal failure affect mortality, and might also increase the risk of cerebral infarction and stroke, and education has been stated to be an important factor in life expectancy and prescription of oral anticoagulation; we therefore adjusted for this potential confounder [18–19]. All potential confounders except age were evaluated on a categorical scale. Age was evaluated on a linear scale with an additional quadratic term if it showed a non-linear relation to outcome. Because of non-proportional hazards, tested on the basis of Schoenfeld residuals, risk time was split at six months and time-dependent models were estimated to separately evaluate one to six months and six to 48 months after diagnosis of AF. Stratified analysis was conducted for men and women combined with CHA_2_DS_2_-VASc score and age at diagnosis: younger than 65, 65–74 and 75–85 years of age. Unadjusted cumulative risk for cerebral infarction and stroke are visualized in figures for patients with CHA_2_DS_2_-VASc score 1. The same strategy of analysis was employed to evaluate the risk for cerebral bleeding of warfarin treatment but, as no evidence of non-proportional hazard was present, only one period, one to 48 months, was evaluated. Measures of associations were hazard ratios (HR) with 95% confidence intervals (95% CI). Statistical analyses were made using SPSS version 22 (IBM Corp., Armonk, NY, USA) or STATA release 14 (STATA Corp, College Station, TX, USA).

## Results

### Baseline characteristics

The mean age was 71.5 ± 11.4 years. The proportion of women was 44.3% and their mean age was higher than in men, 74.1 ± 9.8 versus 69.5 ± 12.1 years, respectively (Table 1). There were 11 535 patients (23.8% women) <65 years, 13 157 (27.2%) 65–74 years and 23 741 (49.0%) 75–85 years. Patients with a 0 point on the CHA_2_DS_2_-VASc score were 3 973 (8.2% of all), 1 point 5 934 (12.3%), 2 points 8 289 (17.1%) and ≥3 points 30 237 (62.4%).

### Cerebral infarction and stroke

**Fig 2:**
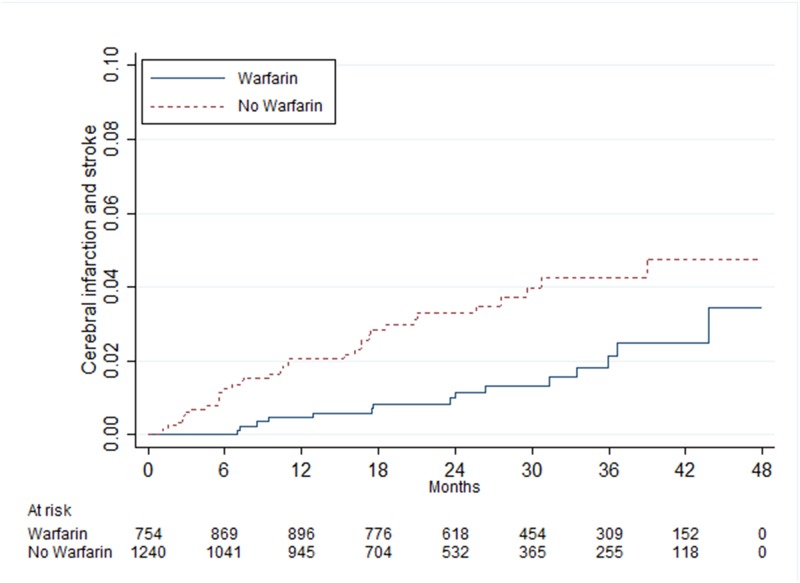
Risk of cerebral infarction and stroke in men 65–74 years of age with atrial fibrillation and one point from the CHA_2_DS_2_-VASc score.

**Fig 3:**
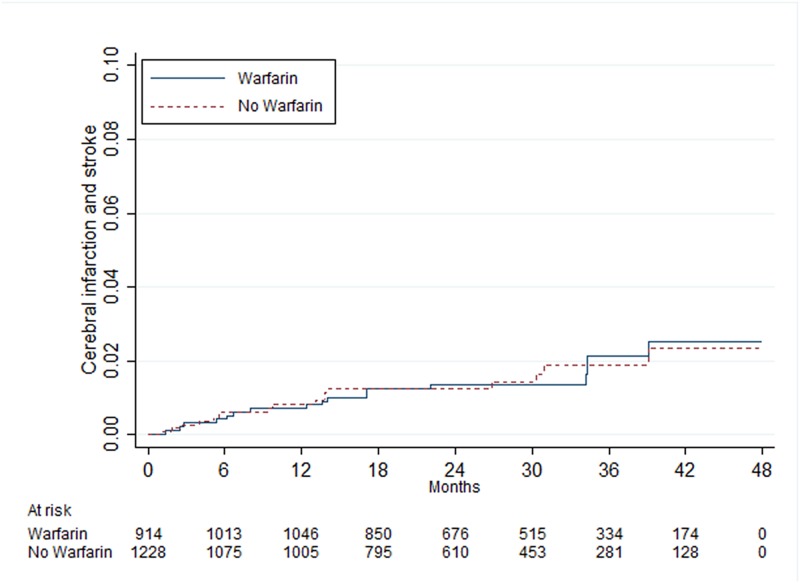
Risk of cerebral infarction and stroke in men <65 years of age with atrial fibrillation and one additional point from the CHA_2_DS_2_-VASc score other than sex or age^a^. ^a^One additional point indicates congestive heart failure, hypertension, diabetes mellitus or vascular disease.

**Fig 4:**
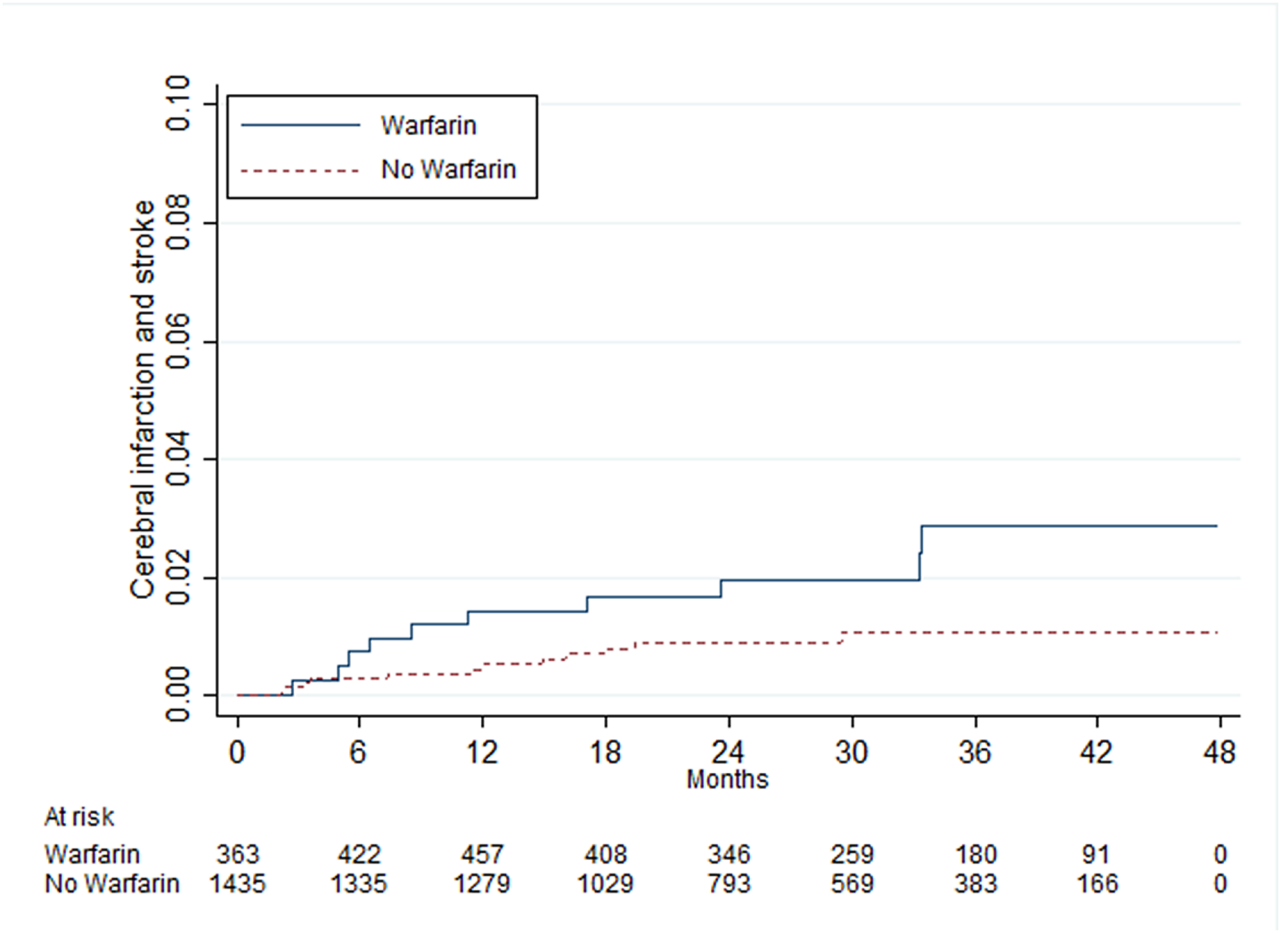
Risk of cerebral infarction and stroke in women <65 years of age with atrial fibrillation and one point from the CHA2DS2-VASc score.

When patients were stratified in groups according to the CHA_2_DS_2_-VASc score (0, 1, 2 and ≥3), age (<65, 65–74 and 75–85) and sex, those with a point of 0 had no statistically significant benefit of warfarin treatment, HR 0.99 (95% CI 0.54–1.80), while patients with ≥3 points had a beneficial effect in all groups, especially in one to six months of follow-up (Table 3). In the group with 1 point, men between 65 and 74 years had a statistically significant benefit of warfarin treatment, HR 0.46 (95% CI 0.25–0.83), while men and women younger than 65 years had no benefit, with HR 1.11 (95% CI 0.56–2.23) and HR 2.13 (95% CI 0.94–4.82), respectively (Figs 2–4). In patients with 2 points, there was a statistically significant benefit of warfarin treatment in all groups except in women younger than 65 years with 1 additional point other than sex as a risk factor.

**Table 3:** Hazard ratios of cerebral infarction and stroke stratified by sex, age and CHA2DS2-VASc score related to time-varying warfarin exposure^a^.

	Warfarin		No warfarin		1–48 months	1–6 months	6–48 months
	N	Events	N	Events	HR (95% CI)^b^	HR (95% CI)^b^	HR (95% CI)^b^
**Men**							
**<65 y**							
**0**	1 426	19	2 890	25	0.99 (0.54–1.80)^c^^,^ ^d^	0.92 (0.23–3.70)^e^	1.09 (0.55–2.16)^c^^,^ ^d^
**1**	1 141	17	1 228	17	1.11 (0.56–2.23)	na	1.07 (0.50–2.33)
**2**	654	11	695	33	0.35 (0.18–0.69)^c^^,^ ^d^	0.07 (0.01–0.52)^c^^,^ ^f^	0.60 (0.27–1.33)^c^^,^ ^d^
**≥3**	440	27	443	59	0.37 (0.23–0.59)^d^	0.27 (0.14–0.52)^c^^,^ ^d^	0.60 (0.28–1.29)^d^
**65–74 y**							
**1**	1 004	15	1 240	36	0.46 (0.25–0.83)^c^	na	0.58 (0.30–1.10)^c^^,^ ^d^
**2**	1 247	25	1 300	57	0.37 (0.23–0.59)^d^	0.14 (0.03–0.64)^c^^,^ ^d^	0.43 (0.25–0.72)^d^
**≥3**	1 822	98	2 062	180	0.53 (0.41–0.68)	0.30 (0.20–0.47)	0.76 (0.54–1.05)
**75–85 y**							
**2**	795	25	1 514	80	0.53 (0.33–0.85)^c^^,^ ^d^	0.40 (0.15–1.07)^c^^,^ ^d^	0.59 (0.34–1.01)^c^^,^ ^d^
**≥3**	3 550	239	6 046	629	0.57 (0.49–0.67)	0.38 (0.28–0.52)	0.67 (0.56–0.80)
**Women**							
**<65 y**							
**1**	538	10	1 435	12	2.13 (0.94–4.82)^c^^,^ ^d^	1.68 (0.33–8.54)^e^^,^ ^f^	2.31 (0.88–6.06)^c^^,^ ^d^
**2**	403	15	589	12	1.84 (0.86–3.94)^d^	na	2.37 (0.97–5.76)^c^^,^ ^d^
**≥3**	382	14	402	38	0.31 (0.16–0.59)^d^	0.11(0.03–0.40)^c^^,^ ^d^	0.76 (0.30–1.94)^f^
**65–74 y**							
**2**	821	13	1 182	42	0.39 (0.21–0.73)^c^	na	0.48 (0.25–0.93)^c^^,^ ^d^
**≥3**	1 948	98	2 039	158	0.58 (0.44–0.75)^d^	0.36 (0.23–0.56)	0.79 (0.55–1.12)
**75–85 y**							
**≥3**	5 096	315	8 777	1 000	0.52 (0.46–0.60)	0.45 (0.35–0.57)	0.56 (0.48–0.66)

na indicates not applicable, too few events; n, numbers; and y, years of age.

^a^Adjusted for age, year of atrial fibrillation, neoplasm, chronic obstructive pulmonary disease, chronic renal failure and education

^b^Hazard ratio (HR) lower than 1 indicates protective effect of warfarin

^c^Due to sparse data, neoplasm, COPD and chronic renal failure were modeled as one co-morbidity; variable coded as yes/no

^d^Due to sparse data, education level 6–7 aggregated

^e^Due to sparse data, neoplasm, COPD, chronic renal failure and education level excluded

^f^Due to sparse data, education level excluded

There were 18 196 patients with a CHA_2_DS_2_-VASc score of 0, 1, and 2 points at the time of inclusion, and, of those, 5 203 patients that increased their score by ≥1 point from risk factors other than cerebral infarction and stroke during follow-up. We separately analyzed the 12 993 patients whose CHA_2_DS_2_-VASc score remained unchanged at 0, 1 and 2 points during the entire follow-up and found the results to be similar to those of the original cohort (Table 4). The message remained that, in patients with a CHA_2_DS_2_-VASc score of 1, only men 65–74 years of age benefitted from warfarin, together with all patients with 2 points, except women <65 years old.

**Table 4:** Hazard ratios of cerebral infarction and stroke in AF patients with an unchanged CHA2DS2-VASc score of 0, 1 and 2 points during the entire study time stratified by sex, age and related to time-varying warfarin exposure^a^.

	Warfarin		No warfarin		1–48 months	1–6 months	6–48 months
	N	Events	n	Events	HR (95% CI)^b^	HR (95% CI)^b^	HR (95% CI)^b^
**Men**							
**<65 y**							
**0**	986	15	2 391	20	1.10 (0.55–2.22)^c^^,^ ^d^	1.23 (0.27–5.65)^e^	1.20 (0.55–2.61)^c^^,^ ^d^
**1**	756	12	887	14	1.15 (0.50–2.65)	na	1.24 (0.48–3.20)
**2**	420	10	477	31	0.37 (0.18–0.77)^c^^,^ ^d^	0.07 (0.01–0.59)^c^^,^ ^f^	0.69 (0.29–1.62)^c^^,^ ^d^
**65–74 y**							
**1**	572	7	837	33	0.31 (0.13–0.72)^c^	na	0.38 (0.16–0.92)^c^^,^ ^d^
**2**	777	19	898	48	0.43 (0.25–0.73)^d^	0.19 (0.04–0.85)^c^^,^ ^f^	0.51 (0.28–0.92)^d^
**75–85 y**							
**2**	553	21	1 092	71	0.54 (0.33–0.91)^c^^,^ ^d^	0.41 (0.14–1.24)^c^^,^ ^d^	0.60 (0.34–1.08)^c^^,^ ^d^
**Women**							
**<65 y**							
**1**	314	7	1 105	11	2.06 (0.83–5.09)^c^^,^ ^d^	1.97 (0.38–10.1)^e^^,^ ^f^	2.14 (0.69–6.62)^c^^,^ ^d^
**2**	242	10	411	11	1.48 (0.65–3.33)^c^^,^ ^d^	na	1.97 (0.71–5.45)^d^^,^ ^e^
**65–74 y**							
**2**	465	6	786	30	0.31 (0.13–0.74)^c^^,^ ^f^	na	0.42 (0.17–1.05)^c^^,^ ^f^

na indicates not applicable, too few events; n, numbers; and y, years of age.

^a^Adjusted for age, year of atrial fibrillation, neoplasm, chronic obstructive pulmonary disease, chronic renal failure and education

^b^Hazard ratio (HR) lower than 1 indicates protective effect of warfarin

^c^Due to sparse data, neoplasm, COPD and chronic renal failure were modeled as one co-morbidity; variable coded as yes/no

^d^Due to sparse data, education level 6–7 aggregated

^e^Due to sparse data, neoplasm, COPD, chronic renal failure and education level excluded

^f^Due to sparse data, education level excluded

### Cerebral bleeding

**Fig 5:**
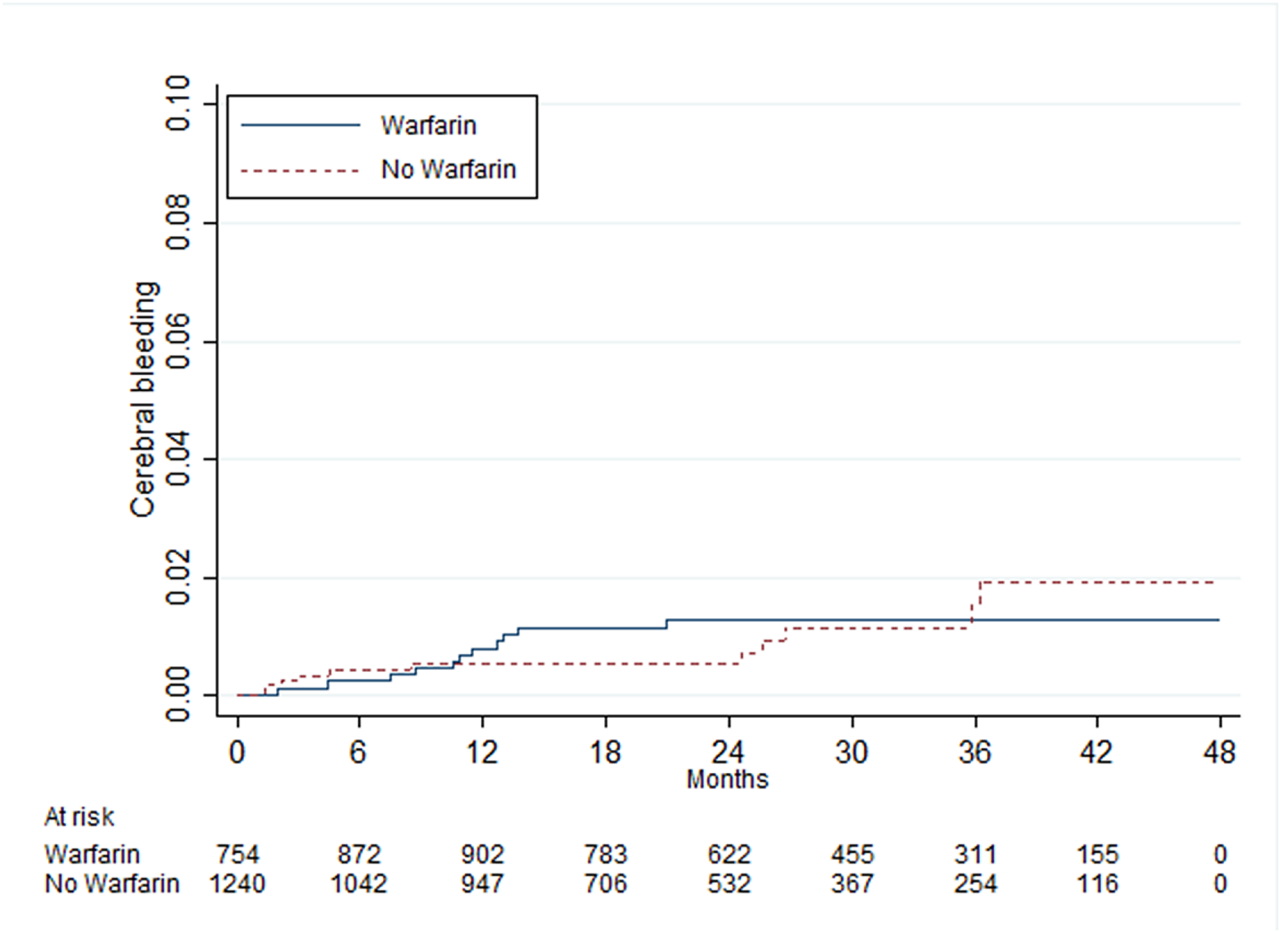
Risk of cerebral bleeding in men 65–74 years of age with atrial fibrillation and one point from the CHA_2_DS_2_-VASc score.

**Fig 6:**
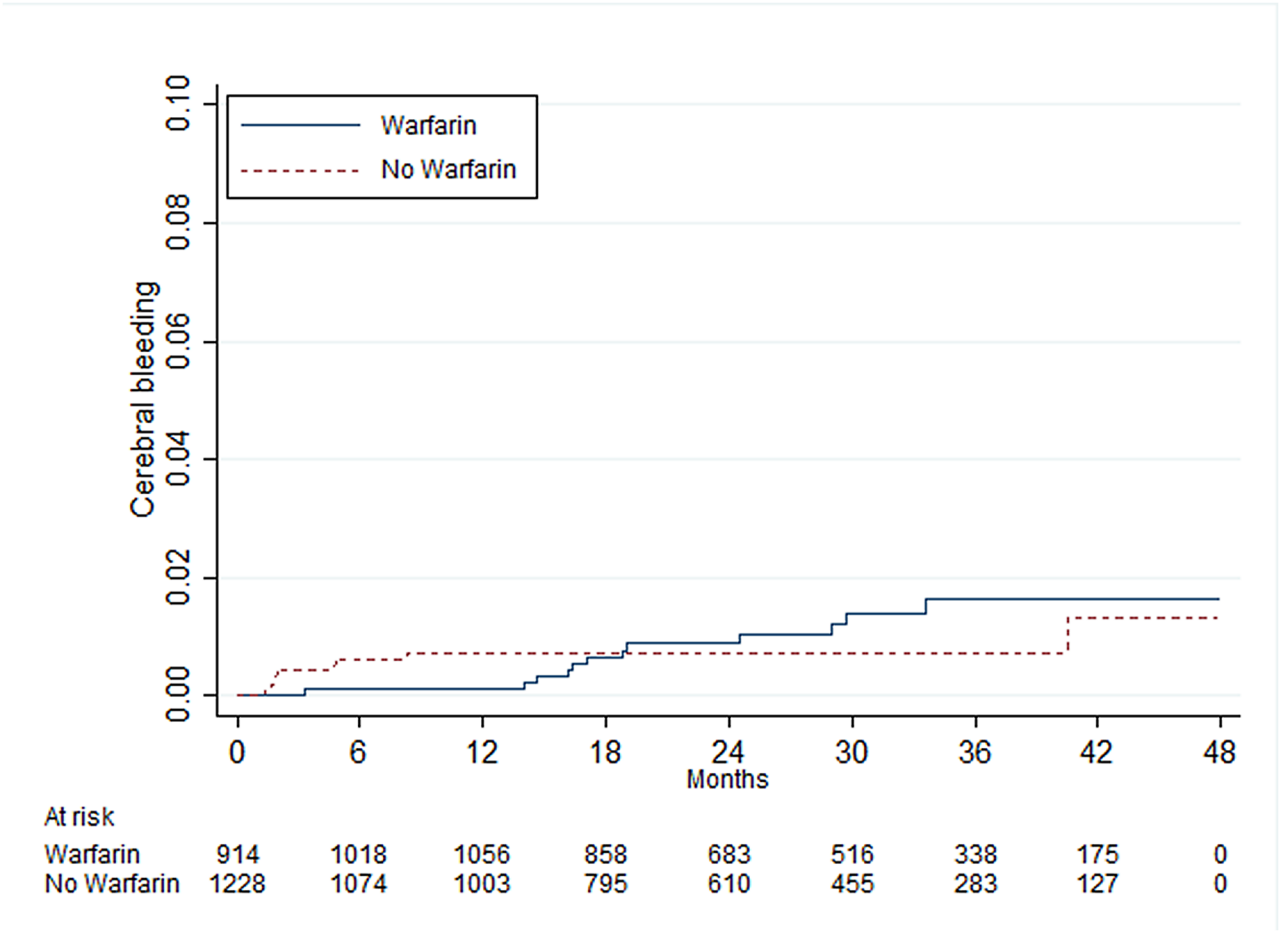
Risk of cerebral bleeding in men <65 years of age with atrial fibrillation and one additional point from the CHA_2_DS_2_-VASc score other than sex or age^a^. ^a^One additional point indicates congestive heart failure, hypertension, diabetes mellitus or vascular disease.

**Fig 7:**
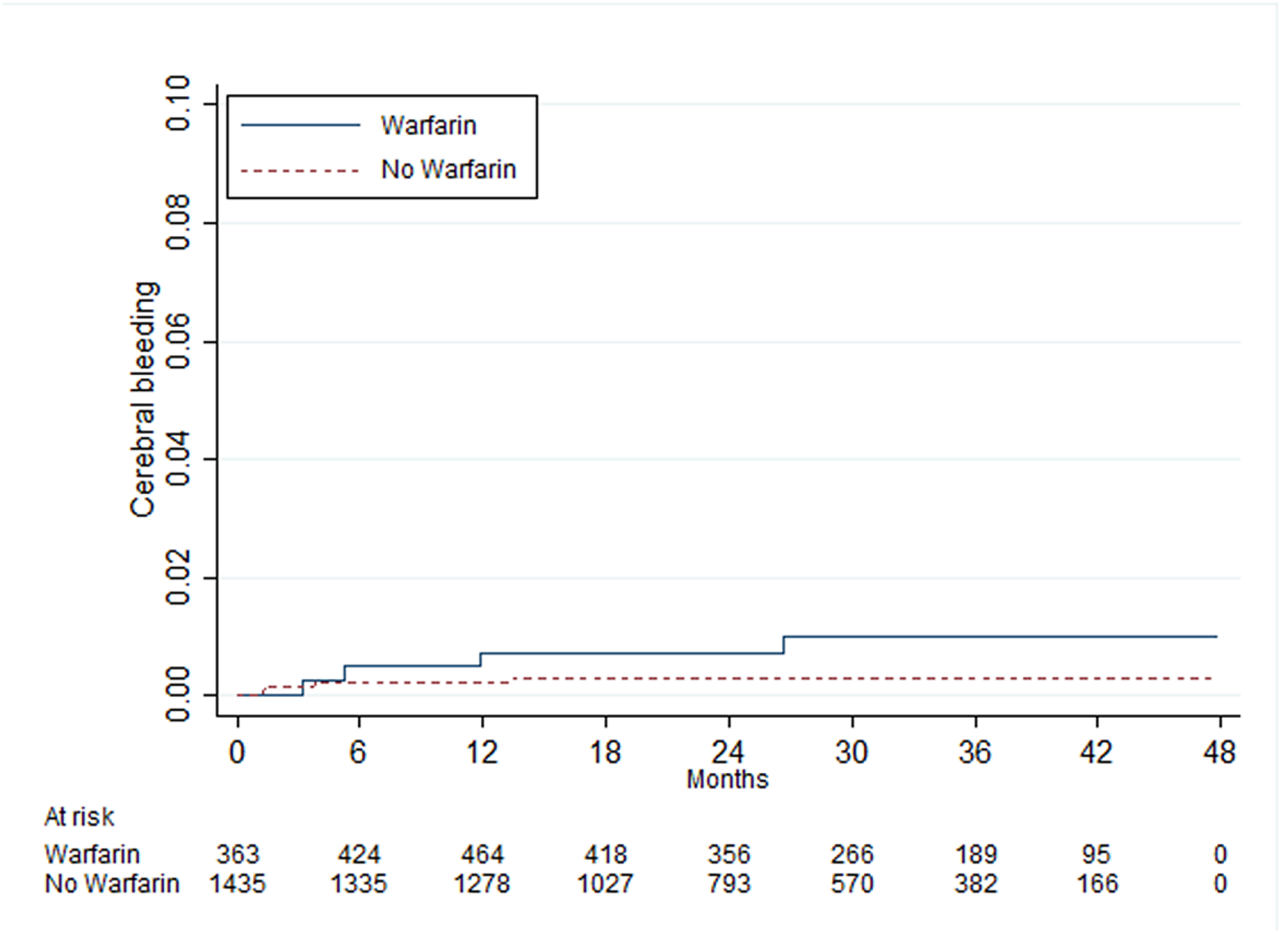
Risk of cerebral bleeding in women <65 years of age with atrial fibrillation and one point from the CHA_2_DS_2_-VASc score.

There was a statistically significant difference between patients with and without warfarin treatment in those with 0 point, where patients with warfarin treatment had an increased risk of cerebral bleeding (Table 5). In patients with 1, 2 and ≥3 points there were no statistically significant differences between those with and without warfarin treatment (Figs 5–7).

**Table 5:** Hazard ratios of cerebral bleeding stratified by sex, age and CHA2DS2-VASc score related to time-varying warfarin exposure^a^.

	Warfarin		No warfarin		1–48 months
	N	Events	N	Events	HR (95% CI)^b^
**Men**					
**<65 y**					
**0**	1 436	10	2 890	4	4.60 (1.40–15.0)^c^^,^ ^d^
**1**	1 151	12	1 228	9	1.53 (0.62–3.78)^c^
**2**	672	5	695	3	1.64 (0.39–6.92)^c^^,^ ^d^
**≥3**	472	8	443	6	0.98 (0.33–2.94)^d^
**65–74 y**					
**1**	1 020	11	1 240	11	1.08 (0.46–2.53)^c^^,^ ^d^
**2**	1 266	16	1 300	13	0.99 (0.47–2.08)^c^^,^ ^d^
**≥3**	1 874	23	2 062	24	0.83 (0.46–1.48)
**75–85 y**					
**2**	810	13	1 514	24	0.77 (0.38–1.55)^d^
**≥3**	3 633	58	6 046	94	0.87 (0.62–1.22)
**Women**					
**<65 y**					
**1**	546	4	1 435	4	2.63 (0.64–10.7)^c^^,^ ^e^
**2**	410	2	589	4	0.64 (0.12–3.53)^e^
**≥3**	398	6	402	7	1.00 (0.31–3.22)^e^^,^ ^f^
**65–74 y**					
**2**	847	5	1 182	5	1.31 (0.37–4.61)^c^^,^ ^e^
**≥3**	2 016	20	2 039	16	1.28 (0.65–2.52)^d^^,^ ^f^
**75–85 y**					
**≥3**	5 254	54	8 777	95	0.89 (0.63–1.27)^d^

na indicates not applicable, too few events; n, numbers and y, years of age.

^a^Adjusted for age, year of atrial fibrillation, neoplasm, chronic obstructive pulmonary disease, chronic renal failure and education

^b^Hazard ratio (HR) lower than 1 indicates protective effect of warfarin

^c^Due to sparse data, neoplasm, COPD and chronic renal failure were modeled as one co-morbidity; variable coded as yes/no

^d^Due to sparse data, education level 5–7 aggregated

^e^Due to sparse data, education level excluded

^f^Due to sparse data, CHA_2_DS_2_-VASc score aggregated 6–9

## Discussion

In this nationwide retrospective cohort study, men and women 65 years and older have a clinically relevant benefit of anticoagulation treatment with warfarin. This also includes otherwise healthy patients, i.e. men with 1 CHA_2_DS_2_-VASc point based on age, and women with 2 points related to age and sex. The prevalence of AF in the population younger than 65 years is 1.2–1.9%; considering the distribution of the total AF population, this corresponds to 18–20% [18,20,21]. This means that approximately 80% of all patients with AF are 65 years or older and might have an indication for treatment with anticoagulants solely based on age as a risk factor.

Risk scores are used to prevent cerebral infarction and stroke while avoiding bleeding complications, and they have different weights on age as a risk factor, from no attention to various points for risks for different age levels [4,22]. We adopted the recommended score, CHA_2_DS_2_-VASc, which includes age categories <65, 65–74 and >75 years giving 0, 1 and 2 points, respectively [4]. In all patients that were 65 years and older, we found a beneficial effect of treatment with anticoagulation, and this is in line with earlier findings that an age between 65 and 74 years carries a strong increase in the risk of cerebral infarction and stroke [5,8,12]. In patients younger than 65 years, we found that those with two additional points from risk factors other than age and sex had a beneficial effect of warfarin treatment which is in line with the current ESC guidelines [3]. There was consistently no obvious beneficial effect of anticoagulation treatment on cerebral infarction and stroke in men and women younger than 65 years and one additional point other than sex in the CHA_2_DS_2_-VASc, which corresponds to women with two points and men with one point. Men and women younger than 65 years had low stroke rates, and, even with an additional risk score point from a disease, the risk of cerebral infarction and stroke remains too low to confer a benefit of anticoagulation. This is in line with earlier findings that hypertension, vascular disease, diabetes mellitus and heart failure increase the risk of cerebral infarction and stroke but that the impact of only one of these conditions is limited [5,12]. In patients with ≥3 points, there was a statistically significant positive effect of warfarin treatment during the first six months after the incident AF as compared to 6–48 months, supporting early initiation of an anticoagulant.

Patients with a low CHA_2_DS_2_-VASc score at inclusion may develop comorbidities during follow-up. This happened among 28.5% of the patients with a CHA_2_DS_2_-VASc score of 0, 1 and 2 at inclusion. Since this study focused on the low risk patients, we separately analyzed the risk of cerebral infarction and stroke in patients who remained at risk score 0, 1 and 2 throughout the study (Table 4). The results in the complete cohort remained and, in patients with a CHA_2_DS_2_-VASc score of 1, warfarin was beneficial only in men 65–74 years of age, while all subgroups benefitted in patients with a score of 2, except women <65 years.

The most severe complication in treatment with warfarin is cerebral bleeding. Our results showed that men younger than 65 years without risk factors and with warfarin treatment had a statistically significantly increased risk of cerebral bleeding. Acetylsalicylic acid in monotherapy shows a similar bleeding risk as warfarin, but the combination increases the risk of bleeding complications and may be a reason for this [3,16]. However, in all other sub-groups, the risk of cerebral bleeding was equal between patients treated or not treated with warfarin. This is consistent with a recent study and indicates that patients with an equal risk set from the CHA_2_DS_2_-VASc score, irrespective of warfarin, have similar risks of intracranial bleeding [23]. Novel oral anticoagulants (NOAC) have shown fewer intracranial bleeding events than warfarin and have been given the same priority as warfarin treatment in the latest guidelines [3]. Our study included only patients on warfarin or not on warfarin because NOAC were not available at that time. However, it is likely that these results as concerns warfarin are directly transferable to NOAC and to all patients with non-valvular atrial fibrillation.

The rates of thromboembolic events vary between study populations, meaning that results of treatment are not necessarily comparable between studies. In a low risk population, 50% of the patients with a CHA_2_DS_2_-VASc of 1 point were 65–74 years old and had an almost significant reduction of stroke with warfarin treatment, but the variants of 1 point were not separately analyzed [24]. Different definitions of the thromboembolic events can at least partly explain differences in annual stroke rates in the placebo groups [5–11]. In comparison, we used as the endpoint cerebral infarction and stroke (I63-I64) and did not include TIA, pulmonary embolus or systemic embolus. We also applied a quarantine period of four weeks to minimize the possibility that the event was not caused by AF, which can have reduced the numbers of endpoints and, finally, we used a time-varying exposure analysis taking into account whether the patients were on active treatment or not.

Age is a risk factor for stroke in several schemes [1–4]. However, the cause of the increased risk of stroke due to age is unknown, but may depend on fibrosis, endothelial dysfunction, inflammatory factors and unmeasured co-morbidities [25]. There are no methods so far to use biochemical markers in a clinical manner for AF patients, but they may be useful in the future [26].

Our findings would have a clinical implication since warfarin is still a common medication. Furthermore, NOAC have shown non-inferiority versus warfarin in preventing thromboembolic events and less intracranial bleeding. For this reason, our results may have relevance when considering any anticoagulant treatment.

## Limitations

Our study is retrospective and includes hospitalized patients only, since subjects who are managed entirely out-of-hospital were not included in the registries. We chose to use a strict endpoint for thromboembolic events, i.e. cerebral infarction and stroke based on diagnoses I63 and I64. Inclusion of TIA and other thromboembolic events might have given other results. We do not know the level of underreporting of comorbidities, especially hypertension, a limitation well known from previous studies and which might lead to a misclassification [5, 6, 7, 19, 23]. On the other hand, a strength of this study is the conclusion of an entire population based on a tax-supported health care system that includes a complete follow-up and validation of patient register have shown a high positive predictive value [13].

Patients with atrial flutter were included since it often coexists in patients with AF, and the electrocardiographic differentiation between atrial flutter and AF can be challenging. We also chose not to differentiate between paroxysmal and sustained forms of AF, well knowing that the type of AF may have affected the choice of anticoagulant treatment at the time, although this would not be a confounder since the risk of stroke is similar [27].

We did not include ASA in the analysis but can not exclude some influence, although studies on ASA and AF did not show any positive or negative effects in the rates of cerebral infarction or cerebral bleeding [3, 15, 16]. Finally, we have no data on the time within the therapeutic range of warfarin treatment, but studies indicate that anticoagulation control in Sweden is satisfactory [28].

## Conclusions

The results of this retrospective study based on hospitalized patients support that warfarin may be considered in all patients with AF and an age of 65 years and older, i.e. also when the CHA_2_DS_2_-VASc score is 1. Our results also support that warfarin is beneficial in patients <65 years of age in men with CHA_2_DS_2_-VASc score ≥2 and in women ≥3 points. In addition, there were no increased risks of cerebral bleeding in these patients.
